# Effects of Anionic Emulsifiers and Emulsified Asphalt on Hydration and Microstructure of Cement

**DOI:** 10.3390/ma17010036

**Published:** 2023-12-21

**Authors:** Panpan Zhang, Yitong Hou, Kaimin Niu, Bo Tian, Hao Wang

**Affiliations:** 1 Research Institute of Highway, Ministry of Transport, 8 Xitucheng Road, Haidian District, Beijing 100088, China; 2 School of Civil Engineering, Chongqing Jiaotong University, Chongqing 400074, China; 3 CCCC First Highway Consultants Co., Ltd., Xi’an 710075, China

**Keywords:** anionic emulsifier, anionic emulsifier asphalt, hydration process, microstructure, delaying effect

## Abstract

Cement-emulsified asphalt (CEA) has been widely used in slab ballastless track and asphalt pavement cold recycling projects because of its high stiffness and toughness. In CEA material, emulsifiers and asphalt affect the cement’s hydration process and microstructure. Thus, to further investigate the effects of anionic emulsifiers (AEs) and anionic emulsified asphalt (AEA) with different demulsification rates on the hydration process and microstructure of cement, two types of AE (rapid-setting and slow-setting) and their corresponding AEA were used to prepare modified cement pastes. First, it was confirmed that the AEs and AEA delayed cement hydration by measuring the setting time, X-ray diffraction (XRD) patterns, and electrical resistivity of the cement paste. Then, the microstructure of the cement paste was determined with mercury intrusion porosimetry (MIP) and a scanning electron microscope (SEM), and it was found that AEs and AEA have varying degrees of inhibitory effects on the formation of the cement paste microstructure. Finally, based on the energy dispersive spectrometer (EDS) element content of the cement paste and Fourier transform infrared spectroscopy (FTIR) on the two AEs, the inhibition mechanism of AE and AEA with different demulsifier rates on the cement hydration process was analyzed. The experimental results showed that both AEs and AEA delayed the hydration process of cement to varying degrees and altered the microstructure of cement, and slow setting anionic emulsified asphalt (SAEA) had the greatest impact on the hydration process and microstructure of cement. Compared to pure cement paste, the initial setting time of cement paste mixed with SAEA was delayed by 73.9%, and the final setting time was delayed by 66.7%. After adding SAEA, the most probable aperture of the cement paste increased from 62.50 nm to 71.19 nm after one day of hydration. Due to the fact that there were more carboxyl groups with negative charges, more -COO^−^ was adsorbed onto the surface of cement particles in the slow-cracking anionic emulsifier (SAE); compared with the rapid-setting anionic emulsifier (RAE) and the rapid-setting anionic emulsified asphalt (RAEA), the SAE and the SAEA had a stronger delaying effect on the hydration reaction of cement.

## 1. Introduction

Cement concrete has been widely used in construction and road engineering due to its excellent compressive strength. However, due to its high elastic modulus, it can also lead to brittle failures. Asphalt concrete has been widely used as a pavement material due to its excellent flexibility and deformation resistance. However, compared to cement concrete, the lower compressive strength of asphalt is an obvious drawback that limits its application range [1]. Researchers have prepared cement-emulsified asphalt (CEA) composites to combine the performance advantages of cement and asphalt materials. CEA is a semi-flexible road-building material composed of cement-based materials and emulsified asphalt. Because CEA has the characteristics of both cement and emulsified asphalt and has high stiffness and toughness, it has been widely used in slab ballastless track and emulsified asphalt cold recycling pavement engineering. Shi et al. [2] prepared a cement emulsified asphalt mortar (CEAM) with good frost resistance using calcium sulfoaluminate cement as raw material. The study found that different cement and emulsified asphalt contents had a significant impact on the microstructure and pore characteristics of CEAM, directly determining its macroscopic properties. This study contributes to the structural formation of calcium aluminate sulfate CEAM and provides a reference plan for the construction of track slabs in winter. Nejad et al. [3] evaluated the improvement mechanism for the mechanical and fatigue properties of CEAM by partially replacing cement with low-pozzolanic activity ground blast furnace slag (GGBFS) and silica fume (SF), providing a theoretical basis for developing more environmentally friendly and better CEAM in high-speed railway construction. Li et al. [4] found that the addition of cement in cold-recycled emulsified asphalt mixture (CRAM) can greatly improve the interface bonding between the binder and filler in the adhesive, thereby enhancing the water loss resistance and high-temperature stability of CRAM.

The addition of emulsifier and emulsified asphalt can affect the hydration reaction of cement in the CEA system, so it is imperative to study the interaction between the cement and emulsifier and emulsified asphalt [5,6]. Li et al. [7] concluded through a setting time test, hydration heat test, and resistivity test on a mixed system of cement and anionic emulsifier asphalt (AEA) that AEA can be selectively adsorbed by cement particles, hindering the deposition reaction of the mineral phase and thus delaying the cement hydration process. Li et al. [8] found that an increase in the amount of cationic emulsifier (CE) leads to a reduction in the chemical-bound water content of the cement paste and the production of Ca(OH)_2_ by measuring the chemical-bound water of the cement and CE mixed system, which proves that CEs delay the hydration behavior of cement. Sun et al. [9] proved through a resistivity test that anionic emulsifiers (AEs) have a lower retarding effect on cement hydration than CEs. Ferrari et al. [10] found that emulsifier and water reducer molecules have a competitive adsorption relationship to cement particles in cement paste through atomic force microscopy, zeta potential, and adsorption measurement tests. Liu et al. [11] believed that compared with AEs and AEA, CE and cationic emulsifier asphalt (CEA) are more easily adsorbed on the surface of cement particles, resulting in a more obvious improvement in the fluidity of cement paste.

In summary, researchers have conducted much research on the interaction mechanism of cement and emulsifiers, as well as of cement and emulsified asphalt, and the influence of emulsifiers on the performance of cement-based materials, but few studies have investigated the impact of AEs and AEA with different demulsification rates on the hydration process and microstructure of cement. AEs and AEA with varying rates of demulsification have different effects on the cement hydration process, which determines their specific application scenarios. For example, in the spraying project, since the CEA needs to be rapidly solidified and hardened in a short time to form strong bonds, and the mixing of cement and emulsified asphalt is not involved in the spraying process, the emulsified asphalt with a poor retarding effect on cement should be selected at this time. In the application of cold recycled mixtures and asphalt pavement base materials, since cement and emulsified asphalt need to be mixed for a long time, emulsified asphalt with a good cement-retarding effect should be selected to increase the workability of the mixture [12,13,14]. Therefore, the selection of emulsifiers is the key step of CEA composite application in engineering [15,16,17,18,19].

The commonly used AEs for preparing AEA include carboxylate, sulfonate, and organic sulfate. Among them, carboxylate emulsifier has high safety and low cost, so most AEA sold on the market is made of carboxylate AE. Based on this, in this study, two kinds of carboxylate AE (rapid-setting and slow-setting) and their corresponding AEA were selected to prepare a cement paste. Setting time, resistivity, X-ray diffraction, mercury intrusion, and scanning electron microscopy tests were used to study the influence of AE and AEA types on the cement hydration process and microstructure, and the influence mechanism of AEs and AEA with different demulsification rates on the cement hydration process was analyzed by measuring the element contents of the cement paste and the infrared spectra of two types of AE. This study fills the research gap regarding the influence mechanism of AEs and AEA with different demulsification rates on cement hydration and microstructure and provides a selection basis for the application of emulsifiers and emulsified asphalt in waterproofing, repair, semi-flexible pavement, and other fields in the future.

## 2. Materials and Methods

### 2.1. Materials

The cement used in this research was ordinary Portland cement P·O 42.5 produced by the Beijing Construction Engineering Group, China. The physical and mechanical properties of the cement were tested in accordance with the Chinese industry standard JTG3420-2020 (MOT, 2020) [18] and are provided in Table 1. The rapid-setting emulsifier and slow-setting emulsifier used in the cement paste were carboxylate anionic emulsifiers produced by Jiangsu Subote New Materials Co., Ltd., China (Nanjing, China). The dosage of both emulsifiers was 0.6% of the cement dosage (mass ratio). The anionic emulsified asphalt was prepared by mixing the two emulsifiers in a ratio of 3% (by mass ratio to emulsified asphalt), and the dosages of both types of emulsified asphalt were 20% of the cement dosage. The water was deionized, and the water–cement ratio was 0.4. The pure cement paste, cement paste containing RAE, and cement paste containing SAE were labelled C, RAEC, and SAEC, respectively. The cement pastes containing the RAEA and SAEA were labelled as RAEAC and SAEAC, respectively. The mix proportions of each group of cement paste are shown in Table 2.

### 2.2. Preparation of Test Samples

Each material was weighted in proportion and the water was poured into the cement paste mixing pot; then, the cement was placed in the mixing pot and the mixer was run at low speed for 120 s and then at high speed for 120 s. At this time, pure cement paste was obtained. Finally, emulsifiers or emulsified asphalt were added to the cement paste, and the above mixing process was repeated to obtain a cement paste mixed with emulsifiers or emulsified asphalt.

### 2.3. Test Method

**Figure 1:**
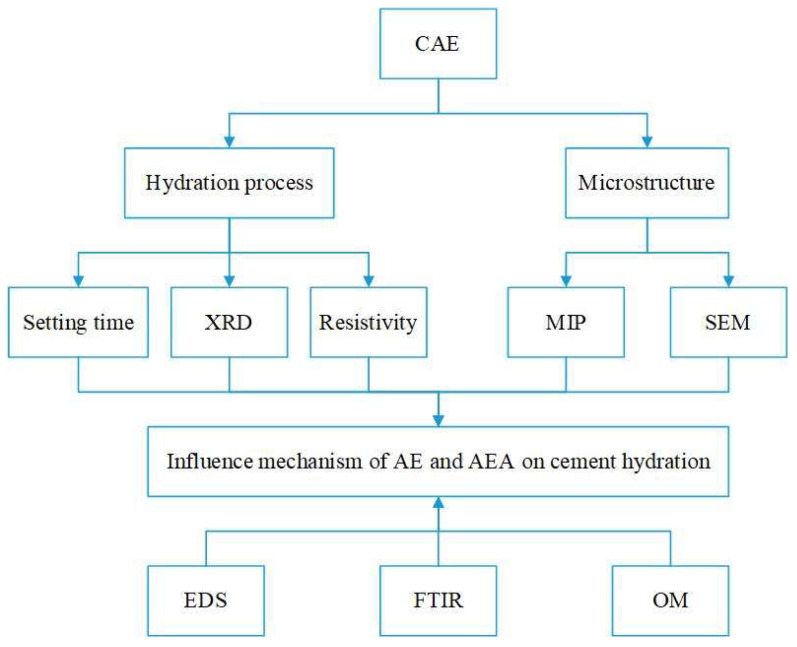
Experimental process.

The influence of AE and AEA types on the cement hydration process was studied from the perspective of macroscopic properties, hydration product formation, and ion dissolution through setting time tests, XRD, and resistivity tests. The influence of AE and AEA types on the microstructure of cement paste was studied through MIP results and SEM results. Based on the EDS elemental spectrum of cement pastes, Fourier transform infrared spectroscopy of two emulsifiers, and optical microscopy images of CAE, the influence mechanism of AEs and AEA on cement hydration process was analyzed. The experimental process is shown in Figure 1.

#### 2.3.1. Setting Time Test

The setting time test for cement paste was carried out following Chinese industry standard JTG3420-2020 (MOT, 2020) [18]. A Vicat apparatus was used to test the setting time of cement pastes. When the cement paste was about to set, the setting situation was observed every 5 min and recorded.

#### 2.3.2. X-ray Diffraction (XRD)

An X-ray diffractometer (Ultima IV, Hitachi, Tokyo, Japan) was used to conduct a continuous in situ test on the hydration products of cement paste after hydration for 4 h. The scanning angle range was 5° to 25°, and the scanning speed was 5°/min.

#### 2.3.3. Resistivity Test

A non-contact electrodeless resistivity tester (CCR-II, Hong Kong, China) was used to monitor the resistivity of the cement paste and to infer the early hydration process of the cement from the perspective of ion dissolution and conductivity.

#### 2.3.4. Mercury Intrusion Porosimetry (MIP)

The pore size distribution and cumulative pore volume of cement paste with an age of 1 d were tested with a fully automatic mercury porosimeter (AutoPore IV 9500, Atlanta, GA, USA) produced by the Mack Company of America. The maximum mercury inlet pressure was 30,000 psia, and the contact angle was 142°.

#### 2.3.5. Scanning Electron Microscope and Energy Dispersive Spectrometer (SEM + EDS)

A scanning electron microscope (Nova Nano 230, Hillsboro, OR, USA) was used to observe the microstructure of the cement paste samples with an age of 1 d. The magnification was 24,000 times, and EDS surface scanning mode was used for micro element analysis.

#### 2.3.6. Fourier Transform Infrared Spectrum (FTIR)

A Fourier infrared spectrometer (Vertex70, Karlsruhe, Germany) was used to conduct an infrared test on the rapid-setting emulsifier and slow-setting emulsifier according to the KBr tablet-pressing method, and the retarding mechanism of different emulsifiers on cement paste was explored based on the characteristic peaks of functional groups.

#### 2.3.7. Optical Microscope (OM)

An optical microscope (DM6 M, Shanghai, China) was used to observe the adsorption process of emulsified asphalt on cement particles. The magnification was 500 times.

## 3. Results and Discussion

### 3.1. Influence of the AE and AEA on the Cement Hydration Process

#### 3.1.1. Setting Time

**Figure 2:**
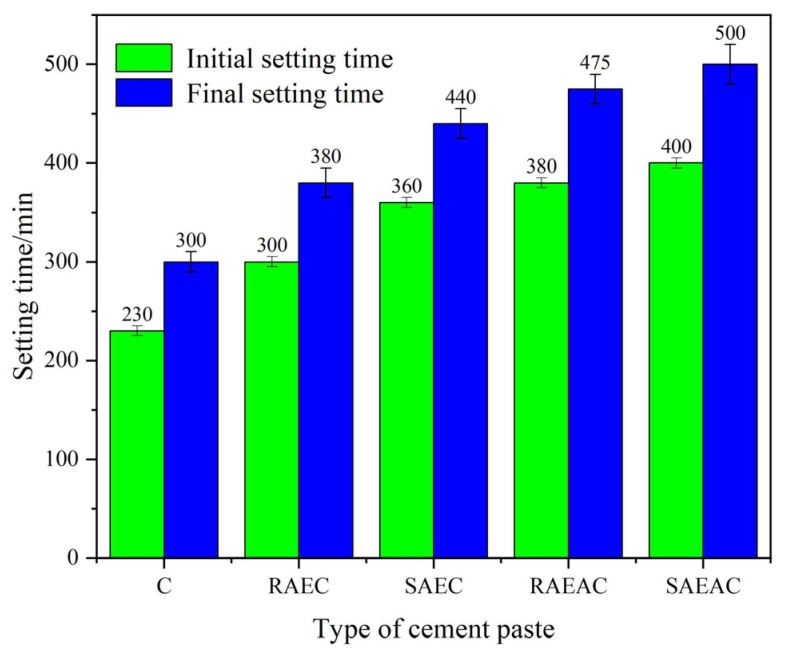
Setting time of cement pastes.

The setting time of cement includes the initial setting time and final setting time. The stronger the cement hydration reaction is, the shorter the setting time is. The influence of anionic emulsifiers on the cement hydration process was preliminarily judged by measuring the setting time of cement. The setting time test results of cement paste are shown in Figure 2.

From the setting time test results, the initial setting times of the RAEC, SAEC, RAEAC, and SAEAC pastes increased by 30.4%, 56.5%, 43.5%, and 73.9% compared with those for C, respectively. The final setting times increased by 26.7%, 46.7%, 58.3%, and 66.7% compared with those of the C paste, respectively. Both AEs and AEA delayed the hydration process of the cement, and AEA had a stronger retarding effect on the cement. This was because the AE was a surfactant, meaning its active groups could adsorb onto the surface of cement particles, changing the surface charge properties of cement particles, increasing the repulsive force between cement particles, and thus slowing down the coagulation speed of cement particles. The influence of AEA on the hydration process of cement was mainly due to the fact that AEA droplets firstly aggregated on the surface of cement particles and compressed to form a layer of asphalt film, which hindered the hydration reaction of cement and prolonged the setting time. This indicated that both emulsifiers and asphalt components hindered the hydration of the cement. Figure 2 also shows that in comparing the effects of AEs and AEA with different demulsification rates on the cement setting times, RAEs and RAEA had a smaller impact on the hydration process of the cement paste, while SAEs and SAEA had a greater delaying effect on the hydration process of the cement paste.

#### 3.1.2. XRD Patterns

**Figure 3:**
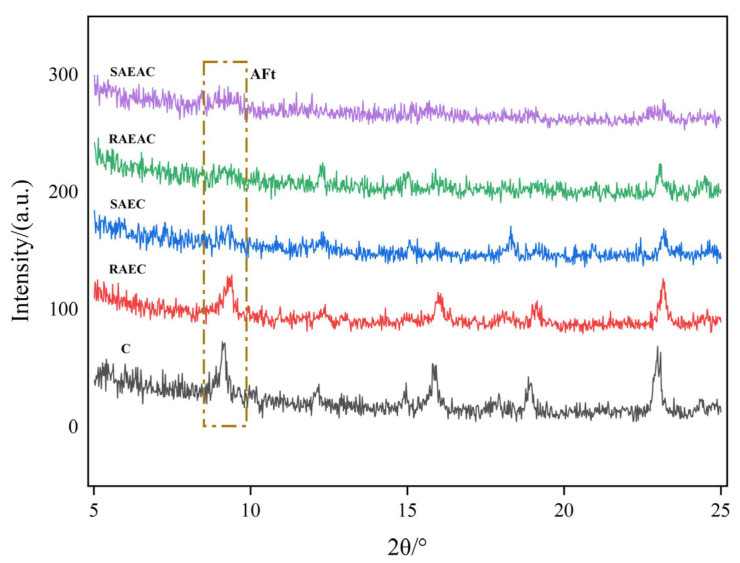
XRD pattern of cement after hydration for 4 h.

Ettringite (AFt) in cement is a crystalline product produced by the combination of cement hydration product C–A–H (hydrated calcium aluminate) and sulfate ions. By measuring the amount of ettringite, the hydration process can be judged from the perspective of the cement hydration products. The XRD patterns of different cement pastes after hydration for 4 h are shown in Figure 3.

The ettringite generated during hydration of the cement paste showed a peak at a 2θ of approximately 8° [20]. Figure 3 shows that SAEC, RAEAC, and SAEAC did not exhibit peaks for ettringite. Both C and RAEC showed peaks corresponding to different contents of ettringite, and the intensity of the characteristic peak for C was slightly higher than that for RAEC. This indicated that after 4 h of hydration, SAEC, RAEAC, and SAEAC had not generated enough ettringite. Large amounts of ettringite were generated in C and RAEC, and the amount of ettringite generated in C was higher than that in RAEC.

When the hydration rate of cement accelerated, the generation rate of hydration products correspondingly increased, meaning the amount of ettringite generated also increased. By comparing the XRD spectra of different cement pastes and the amounts of ettringite generated by each cement paste after 4 h of hydration, it can be concluded that AEs and AEA had a retarding effect on the hydration reaction of the cement, and the degree of delay in cement hydration by SAEs was more significant than that by RAEs. The XRD results validated the reliability of the cement setting time results.

#### 3.1.3. Resistivity

**Figure 4:**
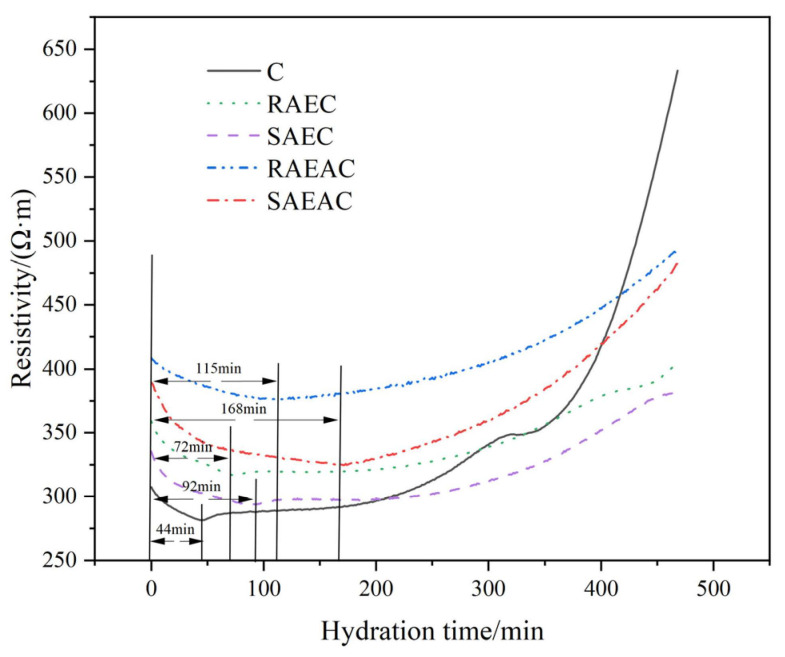
Resistivity curves of cement pastes.

Cement hydrates after encountering water, and ions ionized from the cement mineral clinker are gradually released from the surface of cement particles to the paste. At this time, the cement paste has conductivity due to the free-moving ions [21]. As the cement hydration reaction continues, the ions are recombined freely, thus forming C–S–H, AFt, and other hydration products [22]. At the same time, the water consumption inside the cement paste increases, which shows that the conductivity of the cement paste decreases and the resistivity increases. In the early stage of cement hydration, the more intense the hydration reaction is, the higher the concentration of ions in the solution is, and the internal resistivity of cement paste increases faster over time [23]. The resistivity curves of different cement pastes were tested, and the influence of anionic emulsifiers on the cement hydration reaction process was judged based on the ion dissolution of cement pastes. The resistivity test results are shown in Figure 4.

In the initial stage of cement hydration, the resistivity of the five cement pastes gradually decreased with time because when cement particles came into contact with water various mineral ions were released into the cement paste, making the cement paste an alkaline electrolyte solution with an ionic environment. The ion concentration in the solution gradually increased. Nevertheless, at this time, the ion concentration of the pastes had not reached a particular reaction concentration, and the hydration products were not formed in a large amount. The conductivity of the paste gradually increased with increasing ion concentration; that is, the resistivity of the paste gradually decreased, and the resistivity of the cement paste at this stage was mainly controlled by the ion concentration.

At the same time, the early resistivity of the cement paste mixed with AEs and AEA was higher than that of pure cement paste. The resistivities of RAEC, SAEC, RAEAC, and SAEAC decreased continuously for approximately 72 min, 92 min, 115 min, and 168 min, while the electrical resistivity of C decreased for approximately 44 min. Compared with pure cement paste, AEs and AEA delayed the time when cement hydration entered the formation period of hydration products because AEs and AEA were adsorbed on the surfaces of the cement particles, which inhibited the ion release generated by cement hydration, leading to an ion concentration lower than that of pure cement. Hence, the resistivities of cement pastes with AEs and AEA were higher than that of pure cement paste. Additionally, the continuous decrease in the resistivity of the SAEC lasted 20 min longer than that of the RAEC cement, and the continuous decrease in the resistivity of SAEAC lasted 53 min longer than that of the RAEAC cement. This occurred because more emulsifier was adsorbed onto the cement particles in SAEC and SAEAC.

When the cement hydration ion was released to its ionic solubility product, the resistivity of the five cement pastes increased gradually with time because the products of cement hydration gradually formed the microstructure of the cement paste, which made the resistivity increase gradually. Figure 4 shows that during the acceleration period of cement hydration, the electrical resistivities of the cement pastes mixed with AEs and AEA were significantly lower than that of pure cement paste. The reason for this is that AEs and AEA adsorbed on the surfaces of the cement particles, limited the generation of hydration products, and hindered the formation of the microstructure, resulting in a loose and highly porous paste that provided channels for ion migration and ultimately reduced the electrical resistivity. In addition, the resistivity of the RAEC was always higher than that of the SAEC, and the resistivity of the RAEAC was always higher than that of the SAEAC, indicating that SAEs and SAEA have a stronger retarding effect on the cement paste than RAEs and RAEA.

Combining the setting time test, XRD pattern, and electrical resistivity test results for the cement pastes, it could be seen that both AEs and AEA delayed the hydration process of cement, and SAEs had a stronger retarding effect on cement than RAEs.

### 3.2. Effect of the AEs and AEA on the Microstructure of the Cement Paste

The cement hydration reaction goes through two processes: the process of ion dissolution and the formation process of hydration products. The cement clinker mineral releases a large number of ions into the aqueous solution. At this time, the solution is an alkaline solution containing various ions with strong conductivity. Different ions attract each other to form new hydration products when the ion concentration in the solution reaches the solubility limit. The hydration products overlap and intertwine, creating a microstructure in the cement paste. This microstructure will hinder the movement of ions, leading to the weakening of the conductivity of the cement paste. Therefore, there is a particular internal relationship between the resistivity of cement paste and the microstructure of cement paste. Analyzing the characteristics of cement paste microstructure development is also essential for evaluating the cement hydration process [20].

To intuitively describe the influence of anionic emulsifiers on the microstructure of cement paste, mercury intrusion porosimetry (MIP) and scanning electron microscopy (SEM) were used to observe the microstructure of pure cement paste and cement pastes mixed with AEs and AEA after 1 d of hydration. Thus, the direct internal relationship between the pore structure of cement paste and electrical resistivity was revealed, and the retarding effect of anionic emulsifiers on the hydration reaction of cement paste was further proven.

#### 3.2.1. Characterization of the Pore Structure

**Figure 5:**
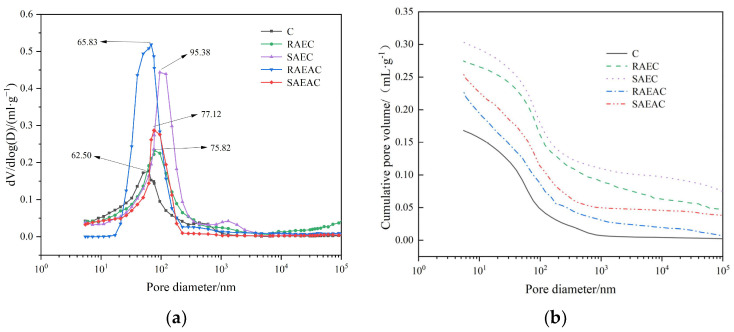
Pore size distribution and cumulative pore volume change curve after cement hydration for 1 d: (**a**) pore size distribution; (**b**) cumulative pore volume curve.

Figure 5 shows the pore size distribution curve and cumulative pore volume of different cement pastes after hydration for one day.

As shown in Figure 5a, the pore size distributions in all five groups of cement had peaks, and the pore size corresponding to this peak was the most probable aperture [24]. This indicates that within this aperture range, the proportion and probability of this aperture were the highest [25]. The most probable apertures for RAEC, SAEC, RAEAC, and SAEAC were 62.50 nm, 75.82 nm, 95.38 nm, 68.53 nm, and 77.12 nm, respectively. Figure 5b shows that the cumulative pore volumes of cement pastes increased in the order C, RAEAC, SAEAC, RAEC, and SAEC. Because the pores in the cement paste came from the original flushing space, which was not filled with hydration products, the differences in porosity and pore volumes for each group of cement were mainly attributed to inconsistent hydration of the cement.

Analyzing the above test results, it was not difficult to see that the addition of AEs and AEA increased the most probable aperture and cumulative pore volume of the cement paste, which indicated that the addition of AEs and AEA inhibited the generation of cement hydration products and delayed the cement hydration reaction. The SAEs and SAEA had a more significant impact in delaying cement hydration than the RAEs and RAEA. In addition, the asphalt components in AEA filled the pores of the cement paste, so the most probable aperture and cumulative pore volumes of RAEAC and SAEAC were smaller than those of RAEC and SAEC.

#### 3.2.2. SEM Images

**Figure 6:**
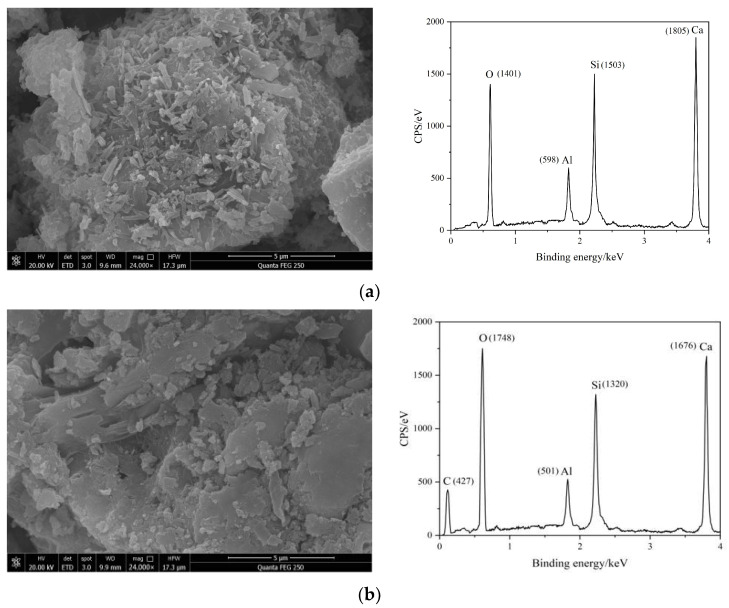

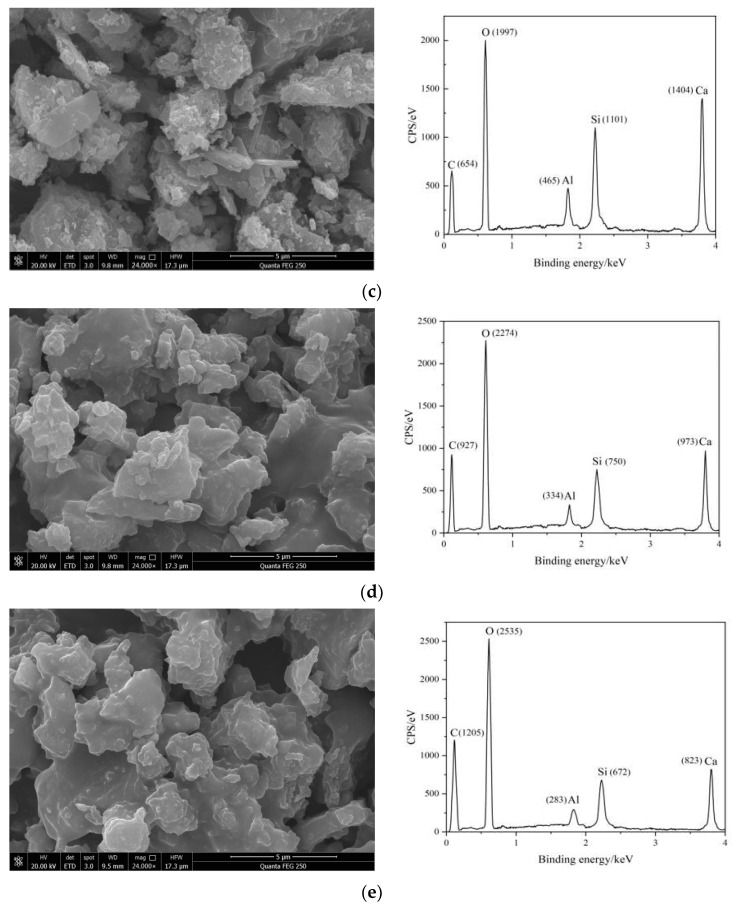
SEM image of cement paste after hydration for 1 d: (**a**) C; (**b**) RAEC; (**c**) SAEC; (**d**) RAEAC; and (**e**) SAEAC.

Figure 6 shows the SEM images and EDS spectra of different cement pastes after one day of hydration.

The SEM images for the different cement pastes clearly show that more hydration products were generated on the surfaces of C. The ettringite particles were slender and had rod-shaped structures, and the structure was very dense. The entire structure had almost no connected pores, indicating that the hydration reaction of C was relatively complete. Although there were also hydration products generated on the surfaces of the RAEC particles, the amount of hydration products generated was less than that of C, and there were small pores and a relatively dense overall structure. However, the surfaces of the SAEC particles had fewer hydration products than those of the C and RAEC, and there were large, connected pores. The overall structure exhibited low strength and a loose shape due to the interpenetration of the large pores. The SEM images of the cement paste mixed with AEA showed that it formed a layer of asphalt membrane on the surfaces of the cement particles, which hindered contact between the surfaces of cement particles and water, thereby delaying the cement hydration reaction and limiting the generation of hydration products. Therefore, there were many pores in the cement paste. However, the asphalt membrane filled the small pores on the surfaces of the cement particles.

The EDS test results for the five sets of cement pastes showed that after the addition of AEs and AEA, the characteristic peaks of C began to form on the cement surface, and the characteristic peaks of O showed varying increases, indicating that the -COO^−^ in AEs and the asphalt component in AEA adhered to the surfaces of the cement particles. The peaks for Al, Si, and Ca decreased to varying degrees, indicating that the adsorption of AEs and AEA on the cement hindered ion dissolution from minerals on the surface of cement, thereby hindering hydration of the cement. Based on the elemental distributions for each group of cement, it was inferred that compared with RAEs and RAEA, SAEs and SAEA had more -COO^−^ groups on the surfaces of the cement particles, which further delayed hydration of the cement.

The SEM images and EDS element maps demonstrated the retarding effect of SAEs on the hydration reaction of the cement paste, and SAEs had a stronger effect than RAEs. Additionally, the SEM images of the cement paste after one day of hydration were consistent with the EDS element analyses and mercury intrusion results.

### 3.3. Analysis of the Retarding Mechanism of the AEs and AEA on Cement Hydration

**Figure 7:**
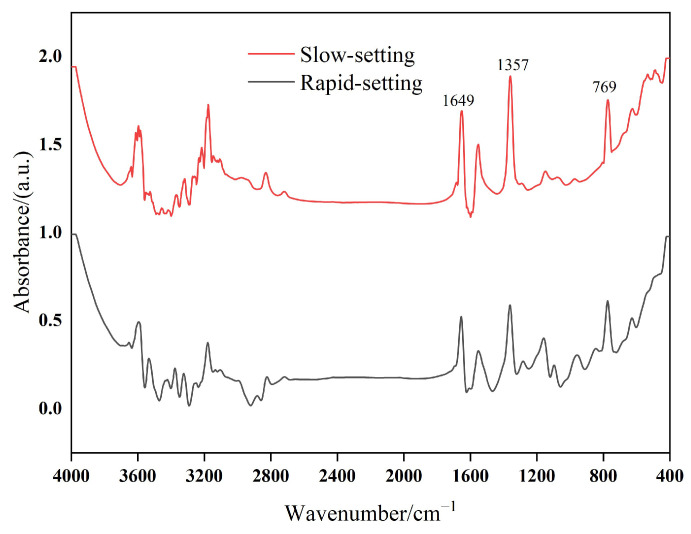
Infrared spectra of different emulsifiers.

By further analyzing the infrared spectrum of emulsifiers, the retarding mechanism of anionic emulsifiers on cement hydration was explored, and the reasons for the difference in the retarding degree of different anionic emulsifiers on cement hydration were analyzed. Figure 7 shows the infrared spectra of different emulsifiers.

In ordinary Portland cement paste, the retarding effect of carboxylate anionic emulsifiers on cement is mainly due to the existence of the hydrophilic group -COO^−^, which can be adsorbed on the surface of positively charged silicate-phase C–S–H and other positively charged ions through electrostatic action. This results in the active site on the surface of cement particles being occupied by anionic emulsifiers, inhibiting the dissolution of surface ions of the mineral phase in the cement paste, thus delaying the hydration process of ordinary silicate cement and hindering the formation of the internal microstructure of the cement paste [6].

According to the infrared spectra of the two emulsifiers, the absorption peak at 1649 cm^−1^ was caused by the C=O stretching vibration in carboxylate; 1357 cm^−1^ was the carboxylate -COO^−^ characteristic peak; and 769 cm^−1^ was the rocking absorption vibration peak in the (CH_2_) n plane. The characteristic peaks of the two emulsifiers were the same, but the intensity of each peak position of the slow-setting anionic emulsifier was significantly higher, indicating that there was more -COO^−^ in the slow-setting anionic emulsifier.

In the early stage of cement hydration, due to the large amount of -COO^−^ in the SAEs and SAEA, -COO^−^ has a relatively large role in the “occupation” of the mineral phase in the ordinary Portland cement paste. This also explains why SAEs and SAEA delay the hydration process of ordinary Portland cement more than RAEs and RAEA.

### 3.4. Analysis of the Hydration Process of Cement Containing AEs and AEA

#### 3.4.1. The Adsorption Process of the AEs on Cement Particles

When cement and water are mixed, the paste undergoes a hydration reaction. Various minerals ionize and release differently charged ions in water. Due to the different migration rates of ions from the surface of cement particles to the aqueous solution, a charged double-layer structure is formed on the surface of cement particles [26,27]. Among them, aluminate-phase C_3_A reacts with water and releases Ca^2+^, OH^−^, and Al(OH)^4−^. When the ion concentration reaches a certain level, the hydrated product AFt is generated, and its surface is positively charged [28]. The silicate phases C_2_S and C_3_S react with water to release a large number of Ca^2+^, OH^−^, and H_2_SiO_4_^2−^ ions. With increasing ion concentrations, some silicate phases complexed with Ca^2+^ to generate a low-calcium–silicon ratio C–S–H(m) gel with a positive charge on its surface [29]. With the progress of cement hydration, the solution reaches a supersaturation state. At this point, the silicate phase precipitates on the surface of the cement particles, and H_2_SiO_4_^2−^, Ca^2+^, and OH^−^ continue to be released. Because the radius of the H_2_SiO_4_^2−^ ion is relatively large, its migration rate to the aqueous solution after dissolution is lower than that of Ca^2+^ and OH^−^, leading to the formation of a “silicon-rich layer” with a negative charge on the surface of the cement particle silicate phase.

**Figure 8:**
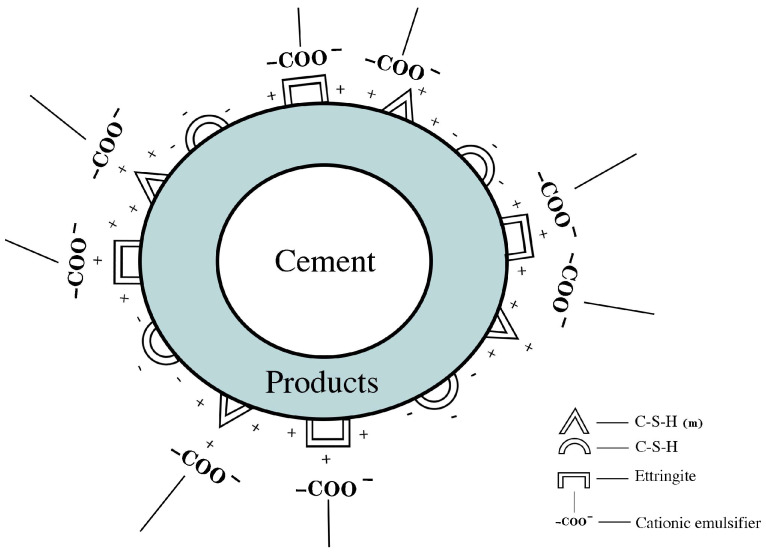
Adsorption of anionic emulsifier on the surface of cement particles.

Figure 8 shows the adsorption process of an anionic emulsifier on cement particles. When an anionic emulsifier is added to the cement paste, the -COO^−^ in the anionic emulsifier is adsorbed on the surface of AFt with a positive charge and part of the C–S–H(m) gel due to electrostatic action, which hinders the deposition reaction on the surface of the mineral phase, and finally delays the cement hydration process, inhibits the formation of cement hydration products, and hinders the development of the microstructure of the cement slurry. The greater the content of -COO^−^ in the anionic emulsifier is, the more obvious its inhibitory effect on the formation of cement hydration products, and the greater the inhibition of the cement hydration process and microstructure development.

#### 3.4.2. Process of Demulsification and Film Formation of the AEA on the Surface of Cement Particles

**Figure 9:**
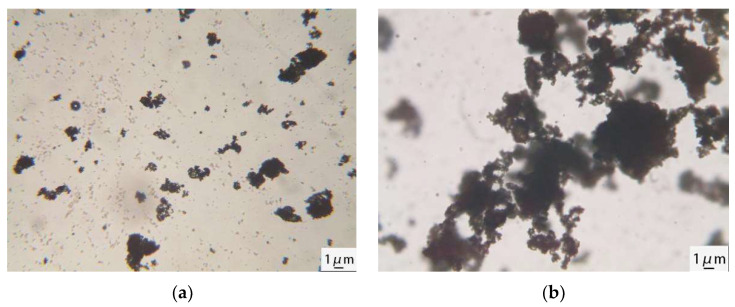
Optical microscope images of cement-emulsified asphalt: (**a**) mixed for 5 min; (**b**) mixed for 20 min.

Figure 9 shows optical microscope images of cement mixed with AEA for 5 and 20 min.

**Figure 10:**
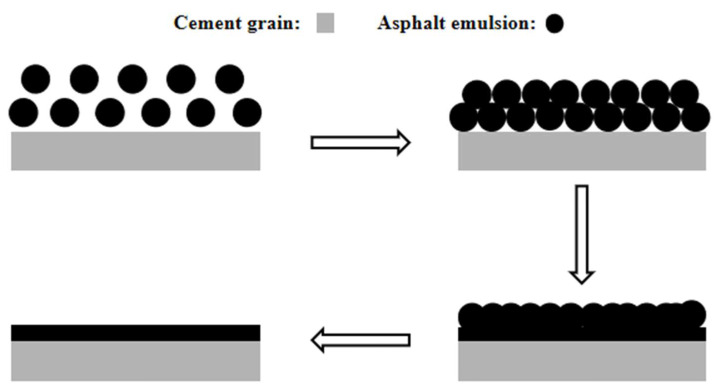
Process of emulsified asphalt demulsification and film formation on the surface of cement particles.

Figure 9a shows that after mixing the cement with AEA for 5 min, the diameters of most emulsified asphalt particles were approximately 1 μm. A few emulsified asphalt particles aggregated to form a flocculent structure. Figure 9b shows that the emulsified asphalt particles were squeezed and fused with each other to form a cross-linked network. This occurred because as the cement hydration reaction proceeded and the hydration products were generated, the AEA particles had a negatively charged double layer [30], and their radii were much smaller than those of the cement particles. They were selectively adsorbed onto the surfaces of the cement particles and hydration products via electrostatic interactions and then squeezed and fused with each other to form large asphalt particles, ultimately accelerating demulsification and forming an asphalt film on the surfaces of the cement particles. The demulsification and film formation processes of the AEA on the cement particles are shown in Figure 10.

Based on the above experimental results, it was concluded that the membrane formed by AEA on the surfaces of the cement particles inhibited cement hydration, but it filled the micropores on the surfaces of the cement particles and improved the microstructure of the cement paste.

## 4. Conclusions

This paper aimed to study the influence of AEs and AEA with different demulsification rates on the cement hydration process and microstructure. By using setting time, XRD patterns, SEM images, and resistivity measurements of the cement paste, the pore structure distribution and the microstructure of the cement paste were observed, revealing the retarding mechanism of AEs and AEA with different demulsification rates on cement hydration. The main conclusions were described as follows:

1. The addition of AEs and AEA increases the setting time of cement slurry and reduces the production of AFt. Among them, the cement slurry with added SAEA had the longest setting time. Compared with pure cement slurry, the initial setting time of the cement slurry with added SAEA was delayed by 73.9%, the final setting time was delayed by 66.7%, and it formed the least AFt, indicating that AEs and AEA delayed the hydration reaction of cement.
2. In the early stage of cement hydration, the electrical resistivity of pure cement paste and cement paste mixed with AEs and AEA gradually decreased over time, and during this stage, the electrical resistivity of cement paste was controlled by the ion concentration. The duration of the decrease in electrical resistivity of cement paste mixed with SAEA was the longest, at 168 min. Because of the adsorption effect of cement particles on AEs, AEs and AEA delayed the time for cement hydration to enter the formation period of hydration products. Due to the difference in the molecular adsorption capacity of emulsifiers, compared with cement pastes mixed with RAEs and RAEA, the hydration process of cement pastes mixed with SAEs and SAEA was slower.
3. The addition of AEs and AEA increased the maximum pore size and cumulative pore volume of cement paste. After adding SAEA, the most probable aperture of cement paste increases from 62.50 nm to 71.19 nm after one day of hydration. SEM images showed that the pores of the cement paste mixed with AEs increased, and there were also large, connected pores in the cement paste mixed with SAEs. The asphalt component in AEA filled the small pores on the surfaces of cement particles. The improvement of this pore structure helps to enhance the mechanical strength of cement. The microstructure of the cement paste showed that AEs inhibited the generation of cement hydration products and delayed the cement hydration reaction. The SAEs had a more significant delaying effect on cement hydration than the RAEs. The EDS test results indicated that the retarding effects of AEs and AEA on the cement mainly involved physical adsorption.
4. The infrared spectra of the two AEs showed that the peak intensity for the carboxylates in SAEs was higher than that in RAEs, indicating that compared with RAEs and RAEA, SAEC and SAEAC have more -COO^−^. This increased the adsorption capacities of the cement particles in the cement paste, resulting in a decrease in ion dissolution from the cement particles and a greater inhibitory effect on the hydration process and microstructure development of the cement.
5. The optical microscopy images of cement-emulsified asphalt paste showed that after mixing of the cement and AEA began, AEA particles aggregated to form a flocculent structure. As cement hydration continued, the AEA particles selectively adsorbed onto the surfaces of the cement particles and hydration products via electrostatic interactions and then squeezed and fused with each other to form large asphalt particles, ultimately accelerating demulsification and forming an asphalt film on the surfaces of the cement particles. This membrane inhibited cement hydration, but it filled the micropores on the surfaces of the cement particles, thereby improving the microstructure of the cement paste.

The selection of emulsifiers and emulsified asphalt is crucial for construction in CEA materials. Choosing appropriate emulsifiers and emulsified asphalt can regulate the rheological properties of CEA, thereby adapting to different construction scenarios and improving construction efficiency. This paper investigated the effects of AEs and AEA with different demulsification rates on cement hydration and microstructure, aiming to provide a theoretical basis for the application of emulsifiers and emulsified asphalt in waterproofing, repair, and semi-flexible pavement fields. Extensive research can be conducted on the application of CEA materials in this field in the future.

## Figures and Tables

**Table 1:** Physical and mechanical properties of cement.

Requirement of Normal Consistency (%)	Setting Time (min)		Flexural Strength (MPa)		Compressive Strength (MPa)	
	Initial Setting	Final Setting	3d	28d	3d	28d
30	180	240	4.1	8.2	21.8	45.2

**Table 2:** Mix ratio of cement paste for each group (mass ratio).

Group	Cement	Water	RAE	SAE	RAEA	SAEA
C	1	0.4	—	—	—	—
RAEC	1	0.4	0.006	—	—	—
SAEC	1	0.4	—	0.006	—	—
RAEAC	1	0.3	—	—	0.2	—
SAEAC	1	0.3	—	—	—	0.2

Note: The solid content of emulsified asphalt in this study was 50%, so the water consumption of cement pastes mixed with emulsified asphalt was the total water consumption minus the water in the emulsified asphalt.

## Data Availability

Data are contained within the article.
